# Bibliographical

**Published:** 1872-03

**Authors:** 


### BIBLIOGRAPHICAL.

Hand-Book of Skin Diseases. By Dr. Isidor Neumann, Docent An Der, K.K. Universitat in Wien. Translated from the Second German Edition, with Notes by Lucien D. Bulkley, A.M., M.D., Surgeon to the New York Dispensary, Department of Venereal and Skin Diseases, etc. Illustrated with Sixty-six Wood-Cuts. New York: D. Appleton & Co., 549 and 551 Broadway.

Dr. Neumann, the author of this book, was for many years Hebra's assistant, and belongs to the school of dermatologists who condemn the prevailing practice of regarding skin diseases as constitutional affections, and has had ample opportunity to prove and confirm the principles announced by Hebra, and adopted by him as the basis of his work.

There is a marked difference in the views of the translator and author, with regard to the practical application of remedies, which we regard as unfortunate; and if the object of the former was, as announced, by giving the views of a large number of observers—especially with reference to their points of difference, rather than their points of agreement—to give such a comprehensive view of dermatology as could be found in no other work, it would seem to have been much more appropriate to have presented such a view in a work of his own; for, while admitting that "Dr. Neumann gives the most correct as well as the newest views and discoveries in the history, etiology, histology and pathology of these diseases," he evidently attaches much more importance to his own convictions in regard to practice, as would appear from the statement: "We can by no means subscribe to the statement on the fifty-sixth page, which is an index to the plan of treatment advised in the whole work, namely—'We place by far the greatest value upon external treatment.' This difference of opinion will, I think, appear abundantly in the notes appended."

This feature of the translator's labors seems the more remarkable from another statement in his preface, namely:—"The plates in the American edition are photographic copies from the original, and introduce us to a new method of the study of these affections—that is, a careful consideration of the microscopic anatomy as the true basis of understanding pathological lesions, without which knowledge, *treatment* must be founded on empiricism." While, therefore, we acknowledge the obligations due to the translator and publisher of so valuable a book as Dr. Neumann's, we cannot regard its merits as at all enhanced by the appended notes and criticism of the author's treatment, as contained in the translator's preface.

ANÆSTHESIA, HOSPITALISM, HERMAPHRODITISM, AND A PROPOSAL TO STAMP OUT SMALL-POX AND OTHER CONTAGIOUS DISEASES. By Sir JAMES Y. SIMPSON, Bart., M.D., D.C.L., late Professor of Midwifery in the University of Edinburgh. Edited by Sir W. G. Simpson, Bart., B.A., Scholar of Gonville and Cain's College, Cambridge. New York: D. Appleton & Co., 549 and 551 Broadway.

This interesting and valuable book contains five hundred and forty-seven pages, two hundred and eighty-seven of which are devoted to Anæsthesia in its various forms and applications, namely:

PART I.—History of Anæsthesia.
PART II.—Defenses of Anæsthesia.
PART III.—The Nature and Power of Various Anæsthetic Agents.
PART IV.—Applications of Anæsthesia in Surgery and Medicine.
PART V.—Applications of Anæsthesia in Midwifery.
PART VI.—Local Anæsthesia.

Over one hundred pages are devoted to Hospitalism, embracing, among other deeply-interesting matters, statistics of mortality in country and hospital amputations, the mortality of amputations as regulated by the size of hospitals, and the degree in which patients are aggregated or isolated. The pages devoted to this subject are of the deepest interest, as will be well understood from a short extract from the article on country amputation statistics, as follows:

"When the two largest hospitals in Scotland—viz., the infirmaries of Edinburgh and Glasgow—were opened in the last century, the buildings of which they then consisted were new and fresh, and comparatively small. In the Edinburgh Infirmary, out of the first ninety-nine cases in which the limbs were amputated, eight of the patients died, or one in twelve; out of the first thirty amputations for disease in the Glasgow Infirmary, one patient only died. At the present day, in these now greatly enlarged and palatial hospitals, the mortality from the same operations has latterly become higher than one in every three operated upon. But surely, during the last fifty or a hundred years, surgery has made signal and striking progress in various ways and various directions.

"The man laid on an operating table in one of our surgical hospitals is exposed to more chances of death than the English soldier on the field of

Waterloo. To render our hospitals as healthy and useful as possible, and in order to acquire sufficient space and air and isolation for their sick inmates, they should be changed from wards into rooms—from stately mansions into simple cottages—from stone and marble palaces into wooden or brick or iron villages.''

These and many other facts and observations make this an exceedingly valuable part of the work.

Some one hundred and fifty pages are devoted to Hermaphroditism, or Hermaphrodism, under which, as a generic term, is discussed: First, some varieties of malformation, in which the genital organs and general sexual configuration of one sex approaches, from imperfect or abnormal development, to those of the opposite; secondly, other varieties of malformation, in which there actually coexist upon the body of the same individual more or fewer of the genital organs and distinctive sexual characters, both of the male and female.

The work is concluded by "A Proposal to Stamp out Small-Pox and Other Contagious Diseases," which has reference not simply to the protective influence of vaccination in regard to the former, but to the "methodic temporary seclusion, segregation or quarantine of those affected with small-pox, until they have completely passed through the disease, and lost the power of infecting and injuring others. *Isolation* is the chief and leading measure required to stamp out small-pox." To this end, he proposes certain systematic regulations, which would doubtless go very far toward the acccomplishment of the desired object, not only as applied to small-pox, but scarlatina, measles and whooping-cough, and other diseases which are communicated from one indiviual to another.

This, like all the writings of the eminent author, is well worthy of a place in the library of every medical man, and, indeed, without some parts of it, a thorough knowledge of the subjects treated could scarcely be had.

TREATMENT OF PULMONARY CONSUMPTION BY HYGIENE, CLIMATE AND MEDICINE, IN ITS CONNECTION WITH MODERN DOCTRINES. By JAMES HENRY BENNETT, M.D., Member of the Royal College of Physicians, London, etc. Second London Edition. New York: D. Appleton & Co., 549 and 551 Broadway.

The first edition of this essay was published in 1866, and was purely clinical. Dr. Bennett states that, although deeply interested in the discussion then going on respecting the intimate nature of tubercle and pulmonary consumption, he thought it best to confine himself to the clinical aspect of the disease. He, therefore, described only the hygienic or sthenic treatment, which gives such infinitely more favorable results than those attained in his younger days. He now declares, however, that such reticence is no longer desirable or possible. Two doctrines are in active conflict—both based on minute histological research, and both exercising a potent influence on the treatment of pulmonary phthisis. The one—that of Virchow and his adherents—contests the tubercular origin of chronic pulmonary consumption, and declares it to be either a new growth, or a mere form of chronic pneumonia. The other—represented by Prof. Bennett, of Edinburgh—discovers and defends the tubercular origin of chronic pulmonary phthisis as connected with an exudation of previously impoverished blood.

The author of this work advocates the views of the Edinburgh Professor, and states as his basis the clinical observation of the disease in question taken by him in 1866, as already referred to. He also refers to the fact that he is sustained in this view by one of the most eminent Americant authors (Dr. Austin Flint), and asserts his belief that most physicians of mature age and enlarged experience, who have investigated the subject, have arrived at similar conclusions with himself, and are indisposed to surrender the term phthisis for chronic pneumonia; while he suggests that most of the advocates of the inflammation origin are young enthusiasts, but inexperienced physicians.

The work discusses the nature and causes of pulmonary phthisis; bodily hygiene, (food, stimulants, exercise, the passions, etc.;) climate and medical doctrines, (warm, temperate, cold, summer, winter, and special localities;) the medicinal treatment of phthisis—the results of modern treatment—prognosis; cases illustrative of the cure and arrest of phthisis; and what are cured consumptives to do in life? and can they marry?

The great central and pervading assertion contained in the book is, that "pulmonary consumption is a curable disease—indeed, in its early stages, often a very curable disease under sthenic or restorative treatment." In proof of the correctness of this statement, he confirms, by mature knowledge, a paper written in 1840, entitled, "Curability of Consumption." At that date, he had just passed a year as one of the resident medical officers of the Salpêtrière, a large asylum hospital in Paris, more especially devoted to aged and infirm women. There he had discovered, in the dead room, in the lungs of many women who had died in advanced life from other diseases, large cretaceous deposits and puckered cartilaginous cicatrices, which proved, beyond a doubt, that they had suffered at one time with tubercular disease, and had spontaneously recovered. This view, as he states, is confirmed by the investigations of various observers since that time, and especially by Prof. Bennett, of Edinburgh, already referred to, who has contributed more largely than any other writer to the establishment of a restorative treatment, the results of which are in the highest degree encouraging.

We most heartily thank Dr. Bennett for his cheerful contribution to the literature of this fell destroyer, and earnestly recommend it to our readers.

We have received from Henry C. Lea, Publisher, Philadelphia, and will notice in the next number of the JOURNAL—

THE PRINCIPLES AND PRACTICE OF SURGERY. By JOHN ASHHURST, Jr., M.D., Surgeon to the Episcopal Hospital; Surgeon to the Children's Hospital, etc. Illustrated with Five Hundred and Thirty-Three Engravings on Wood.

AN INTRODUTION TO PATHOLOGY AND MORBID ANATOMY. By T. HENRY GREEN, M.D., London, Member of the Royal College of Physicians; Lecturer on Pathology and Morbid Anatomy at Charing Cross Hospital Medical School, etc. Illustrated by Numerous Engravings on Wood.

A TREATISE ON HUMAN PHYSIOLOGY. Designed for the Use of Students and Practitioners of Medicine. By JNO. C. DALTON, M.D., Professor of Physiology and Hygiene in the College of Physicians and Surgeons, New York, etc., etc. Fifth Edition, Revised and Enlarged, with Two Hundred and Eighty-Four Illustrations.

PULMONARY CONSUMPTION: ITS NATURE, VARIETIES, AND TREATMENT. With an Analysis of One Thousand Cases to Exemplify its Duration. By C. J. B. WILLIAMS, M.D., F.R.S., Fellow of the Royal College of Physicians, Senior Consulting Physician to the Hospital for Consumption, Brompton, etc., and CHAS. THEODORE WILLIAMS, M.A., M.D., Oxon, Fellow of the Royal College of Physicians, and Physician to the Hospital for Consumption, Brompton.

